# Synthesis of Glycosides by Glycosynthases

**DOI:** 10.3390/molecules22091434

**Published:** 2017-08-30

**Authors:** Marc R. Hayes, Jörg Pietruszka

**Affiliations:** 1 Institut für Bioorganische Chemie, Heinrich-Heine-Universität Düsseldorf im Forschungszentrum Jülich, 52426 Jülich, Germany; m.hayes@fz-juelich.de; 2 Forschungszentrum Jülich, IBG-1: Biotechnology, 52426 Jülich, Germany

**Keywords:** biocatalysis, glycoside, polysaccharide, glycosidase, glycosynthase, synthesis, glycosylation

## Abstract

The many advances in glycoscience have more and more brought to light the crucial role of glycosides and glycoconjugates in biological processes. Their major influence on the functionality and stability of peptides, cell recognition, health and immunity and many other processes throughout biology has increased the demand for simple synthetic methods allowing the defined syntheses of target glycosides. Additional interest in glycoside synthesis has arisen with the prospect of producing sustainable materials from these abundant polymers. Enzymatic synthesis has proven itself to be a promising alternative to the laborious chemical synthesis of glycosides by avoiding the necessity of numerous protecting group strategies. Among the biocatalytic strategies, glycosynthases, genetically engineered glycosidases void of hydrolytic activity, have gained much interest in recent years, enabling not only the selective synthesis of small glycosides and glycoconjugates, but also the production of highly functionalized polysaccharides. This review provides a detailed overview over the glycosylation possibilities of the variety of glycosynthases produced until now, focusing on the transfer of the most common glucosyl-, galactosyl-, xylosyl-, mannosyl-, fucosyl-residues and of whole glycan blocks by the different glycosynthase enzyme variants.

## 1. Introduction

With the increasingly growing knowledge of the major role glycosides and glycoconjugates play in biological processes, the demand for simple methods for the synthesis of defined glycosides is constantly rising. Their high biological functionality and complex structure is a result of their numerous functional moieties, diverse stereochemistry, and numerous linkage possibilities. Glycosidic structures are ubiquitous throughout Nature, covering the surface of all cellular organisms and even the macromolecules inside these. Therefore, glycosides fulfill many different functions in biological systems providing on the one hand, function, structure and stability for cells and enzymes, but also acting as recognition motifs, as for example, in blood group antigens. Many bacterial and viral infections are mediated by glycoside recognition, clearly demonstrating the importance of glycosides for the pharmaceutical industry [1]. Additionally, specific oligosaccharides, such as human milk oligosaccharides have been described to have probiotic and antimicrobial effects making them highly desired targets for the food and nutrition industry [2]. Polysaccharides also play an important role in discovering new sustainable materials as they originate from renewable resources. Synthesis of glycoside structures would therefore greatly improve the possibility in producing new pharmaceuticals and materials, and allow targeted research on functional and structural properties of these compounds.

**Scheme 1:**
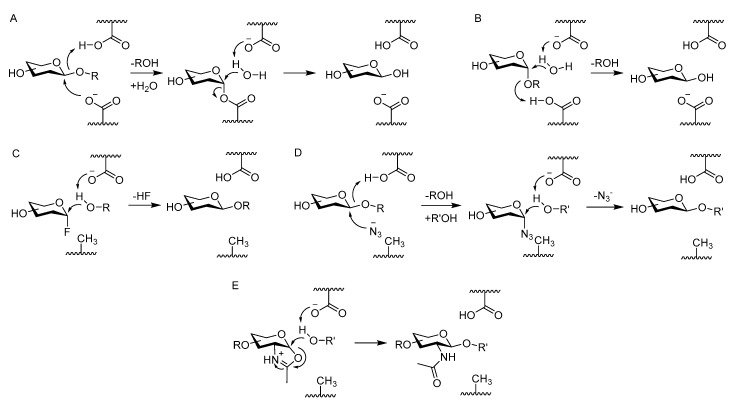
Mechanisms of glycosyl hydrolases and synthases. (**A**) Retaining hydrolases follow a double displacement mechanism in which the enzyme forms a glycosyl-enzyme-intermediate by attacking the anomeric centre with a nucleophilic amino acid residue. The anomeric configuration of the substrate is retained in the product; (**B**) Inverting hydrolases follow a single displacement mechanism, which is supported by acid/base residues. The anomeric configuration of the product is inverted compared to the substrate; (**C**) ‘Classic’ glycosynthase mechanism involving an activated glycosyl donor in opposite configuration to the natural substrate. The product cannot be hydrolyzed due to the lack of a nucleophilic residue; (**D**) Alternative glycosynthase mechanism producing the activated donor in situ with an external nucleophile (azide, formate or acetate ion); (**E**) Glycosynthases derived from *endo*-β-*N*-acetylglucosaminidases transfer oxazoline glycoside donors to variable acceptors.

The chemical synthesis of glycosides is well developed, but remains a laborious task, as many protection and selective deprotection steps are required to solely address single functional groups in order to avoid side product formation [3,4,5,6]. Complete anomeric control of the newly formed bond is also a prerequisite to avoid lengthy separation methods. Throughout nature the synthesis of glycosidic structures is accomplished by the enzyme group of glycosyltransferases. These enzymes transfer activated sugar nucleotide donor molecules selectively onto an acceptor molecule. Their use in biocatalysis has been extensively researched, but is still limited for a large scale by the high cost of the nucleotide-phosphate donors and difficulty in expression and handling of these mostly membrane bound enzymes [7]. Alternative carbohydrate active enzymes are glycohydrolases or glycosidases, of which a high variety are readily available due to their occurrence in the metabolic pathways of all organisms [8]. This group of enzymes naturally degrade glycosidic structures and are defined into two groups depending on their catalytic mechanism (Scheme 1A,B) [9]. Retaining glycosidases follow a double displacement catalyzed by a nucleophilic and acid/base residue in the enzymes active site resulting in a retention of the anomeric configuration of the substrate in the yielded product. Inverting glycosidases in comparison follow a single displacement mechanism with two catalytic acid/base residues supporting the nucleophilic substitution at the anomeric center of the substrate. Glycosidases can be utilized in synthetic reactions by reverse hydrolysis or transglycosylation methods [10,11]. Nevertheless, the ability of the glycosidase in hydrolyzing the produced product leads to strongly diminished yields. To overcome the disadvantage of product hydrolysis Mackenzie et al. and also Malet et al. (working on *exo*- and *endo*-glycosidases, respectively) reported the production of genetically engineered glycosidases, namely glycosynthases, which were lacking a nucleophilic residue and therefore void of hydrolytic activity [12,13]. However, the intact structure of the enzyme allowed the formation of glycosidic bonds in high yields in the presence of activated glycosyl donors such as glycosyl fluorides (Scheme 1C).

Since the introduction of this ‘classical’ glycosynthase method mostly restricted to retaining glycosidases, alternative methods such as in situ generation of donor molecules have been reported, expanding the repertoire of glycosynthases greatly [14]. Glycosidases of various origins have been transformed into glycosynthases enabling the production of α- and β-glycosyl linkages with glucosyl, galactosyl-, fucosyl-, arabinosyl-, mannosyl- or even lactosyl-residues [15]. Most recently, the method has been transferred to *endo*-β-*N*-acetylglucosaminidases, such as Endo-M of *M. hiemalis,* which play important roles in protein post-translational glycosylation. Elucidation of the mechanistic properties of these enzymes led to the development of synthase-like mutants, which transfer oxazoline glycoside structures onto acceptors enabling the *en bloc* transfer of large glycans [16]. This has resulted in a powerful tool for modifying peptides with high pharmaceutical interest with defined glycan structures.

There are many excellent reviews on the topic of enzymatic glycoside synthesis, which give a great overview of the biocatalytic methods but manly focus on the production and mechanistic studies of glycosynthases [10,17,18,19]. This review will focus on the synthesis of glycosides employing glycosynthase methods, only, summarizing in each section the findings of a specific glycosyl residue also giving an overview of described products, which have been synthesized, rather than focusing on the production of the glycosynthase itself.

## 2. Glycoside Syntheses Using Glycosynthase Methods

### 2.1. Glucosynthases

Since the introduction of the genetically engineered glycosynthases by Mackenzie et al. in 1998, glucosynthases derived from glucosidases (*exo* or *endo*) originating from a variety of organisms have been the most commonly produced type of these enzymes [12]. This type of variant is also often named generally ‘glycosynthase’ throughout literature, due to the often-observed promiscuity in regard to the donor glycoside. The Abg E358A glycosynthase, for example, catalyzed not only the transfer of glucosyl but also galactosyl residues to *para*-nitrophenyl (pNP) glycosides. Further modulation of this enzyme broadened the scope of donor substrates as reported by Shim et al. and Kim et al. [20,21]. The use of the glycosynthase variant Abg NNT was thereby extended to C3-modified (methylated) gluco- and galactosyl donors (producing β-1,4 linkages) and Abg 2F6 to a xylosyl donor (Section 2.3), giving a much broader range of synthesizable glycosides. The promiscuity of glycosynthases to accept different glycosyl fluoride donors was also impressively demonstrated by Wei et al. with the β-glycosidase mutant TnG E338A of *T. nonproteolyticus* [22]. The glycosynthase showed high variability towards the donor molecule, transferring α-d-glucosyl, α-d-galactosyl, and α-d-fucosyl fluoride donors (**1**, **2** and **3**) to different acceptors **4**–**8** in yields for **9**–**13** varying between 15–100% depending on the donor and acceptor combination (Table 1). Interestingly the enzyme also exhibited activity towards dihydroisoandrosterone (**5**), an unusually large, lipophilic acceptor, with α-d-glucosyl fluoride (αGlcF, 1) as the donor glycoside (49% yield). An even broader donor scope was exhibited by the glycosynthase BGlu1 E386G originating from the β-glucosidase of rice examined by Pengthaisong et al. [23]. The glycosynthase was shown to transfer glycoside residues of α-d-glucosyl (**1**), α-d-galactosyl (**2**), α-d-fucosyl (**3**), α-d-arabinosyl, α-d-xylosyl (**14**), and α-d-mannosyl (**15**) fluoride donor to pNP-cellobioside **8** producing yields of 57%, 59%, 42%, 99%, 3% and 79%, respectively.

**Scheme 2:**
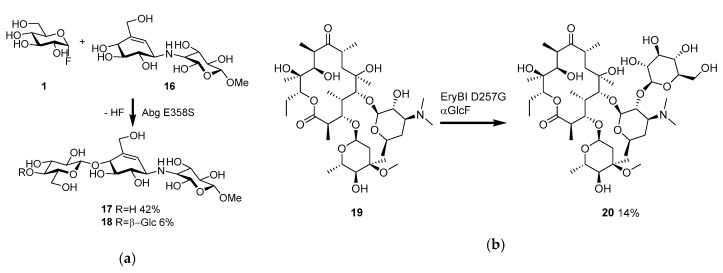
Glucosylation of natural and unnatural glycosides. (**a**) Synthesis of glucosylated methyl β-acarviosin (**16**) by Fairweather et al. [24]. The mono- and diglycosylated products **17**, **18** were isolated in yields 42% and 6%; (**b**) Glucosylation of erythromycin A (**19**) by the glucosynthase EryBI D257G [26]. The enzyme showed high tolerance towards the bulky dimethylated amino group in the C3 position.

The use of the Abg E358S glycosynthase in the synthesis of natural or unnatural glycosides was also demonstrated efficiently by Fairwether et al. in the synthesis of glycosylated versions of methyl β-acarviosin (**16**), an inhibitor of cellulases (Scheme 2a) [24]. The inhibitor was glucosylated (β-1,4) to the tri- **17** and tetrasaccharide **18** in yields of 42% and 6%, respectively.

Further putative cellulase inhibitors, variants of isofagomine and tetrahydrooxazine were produced by MacDonald et al. producing a mixture of cello-oligosaccharide inhibitor variants with different lengths [25]. The reported glucosylation of erythromycin A (**19**) by Jakeman et al. showed the possibility of transferal of a glucosyl residue (β-1,2) to a more complex type of acceptor in a yield of 14% (**20**, Scheme 2b) [26].

**Scheme 3:**
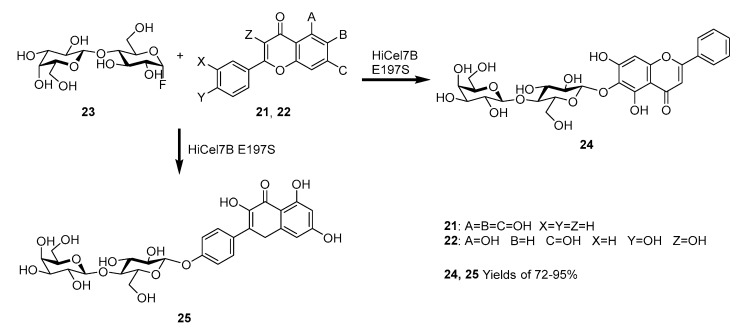
Direct glycosylation of flavonoids **21** and **22** utilizing the glycosynthase HiCel7B E197S [30]. The enzyme exhibited high specificity for glycosylation of the hydroxyl function in the Y position (as for flavonoid **22**) only deviating in the case of absence of this group as observed for the flavonoid **21**. The yields of the flavonoid glycosylation ranged from 72–95%.

In addition to the ‘classical’ glycosynthase approach catalyzing the transfer of glycosyl fluoride donors, Pozzo et al. improved glycosylation yields by utilizing the chemical rescue method for in situ donor production with the β-glucosynthase (TnBgl1A E349G; GH1) of *T. neapolitana* [27]. The group achieved a 3.7× higher yield during the synthesis of quercetin-3,4′-di-*O*-β-d-glucopyranoside with *ortho*-NPGlc and formate for in situ donor production, compared to the synthesis using αGlcF (**1**, 10% yield). This approach was subsequently transferred efficiently to the TnBgl3B D242A W243F (GH1; *T. neapolitana*) glycosynthase which exhibited transglucosylation activity to quercetin-3-*O*-β-d-glucopyranoside (30%), quercetin-3-*O*-β-d-galactopyranoside (40%), and quercetin (15%) [28]. The chemical rescue method was also applied to the α-glucosynthase variant of AglA from *T. acidophilum* (AglA D408G) enabling the synthesis of the putative tyrosinase inhibitor α-1,4-d-glucosyl arbutin in 38% yield [29]. Though most *exo*-glycosidase derived glycosynthases are limited to glycosyl transfer to glycosides, Yang et al. utilized the glycosynthase of HiCel7B, HiCel7B E197S in the glycosylation of non-glycosylated flavonoid structures (e.g., **21** and **22**, Scheme 3) [30]. The former *endo*-cellulase catalyzed the transfer of lactosyl fluoride (**23**, LacF) with high selectivity to the 4′-*O* position (position Y) of four different flavonoids with yields ranging between 72–95% (**24**/**25**, Scheme 3).

A different variant of this enzyme, HiCel7B E197A was recently employed in the synthesis of regularly substituted, functionalized celluloses by Codera et al. [31]. Polymerization of 6′-azido-α-cellobiosyl fluoride by HiCel7B E197A resulted in regularly azido substituted cellulose oligosaccharides with a degree of polymerization (DP) up to 32 in a yield of 88%. A subsequent copper catalyzed azide/alkyne cycloaddition (Huisgen reaction) with alkyne functionalized Alexa Fluor die led to fluorescent polymers. The synthesis of a further polysaccharide was performed by Aragunde et al. [32]. The glycosynthase produced from the β-1,3-1,4-glucanase of *B. licheniformis* catalyzed the oligomerization of the Glc-β1,4-Glc-β1,3-GlcαF donor reaching yields of 90% (weight polymer/weight initial donor).

### 2.2. Galactosynthases

**Scheme 4:**
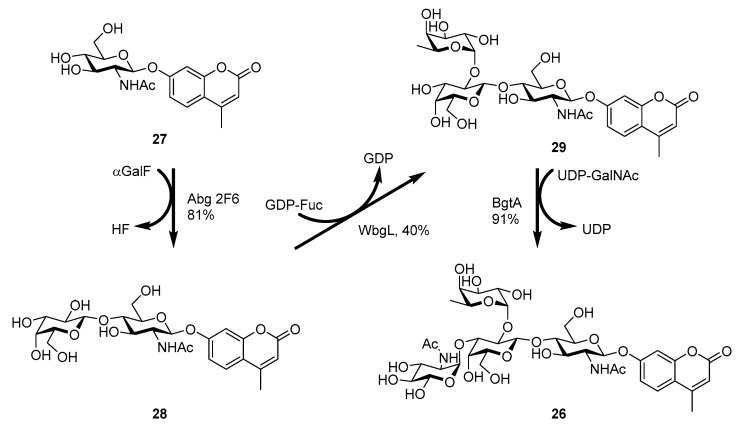
Synthesis of the methyl umbelliferone derivative of the type 2 blood group A oligosaccharide **26** performed by Kwan et al. [33]. The synthesis combined the engineered glycosynthase Abg 2F6 (A19T, E358G, Q248R, M407V) with two glycosyltransferases WbgL and BgtA. The glycosyltranferases could also be employed in a one-pot reaction giving a higher yield of 62% compared to the total yield of 36% for the sequential reaction.

Showing the importance of the glycosynthase method in the production of glycoside structures for biological or pharmaceutical research, Kwan et al. described the synthesis of the type 2 blood group A oligosaccharide **26** by combining the glycosynthase Abg 2F6 in a reaction sequence with glycosyltranfersases WbgL and BgtA (Scheme 4) [33]. The enzyme efficiently transferred α-d-galactosyl fluoride (**2**, αGalF) onto methyl umbelliferone β-*N*-acetylglucosamine (**27**, UM-β-GlcNAc) in yields of 84% (**28**). By exploitation of the chemical rescue method (Section 1, Scheme 1D) usually carried out in the case of α-glycosynthases, Strazulli et al. catalyzed the formation of various β-galactosyl glycosides by the β-galactosynthase AaβGal D361G (*A. acidocaldarius*) and simple donor pNPGal as starting material [34]. Though a high transglycosylation was achieved, the enzyme showed high promiscuity producing different regioisomers and polyglycosylated products.

**Scheme 5:**
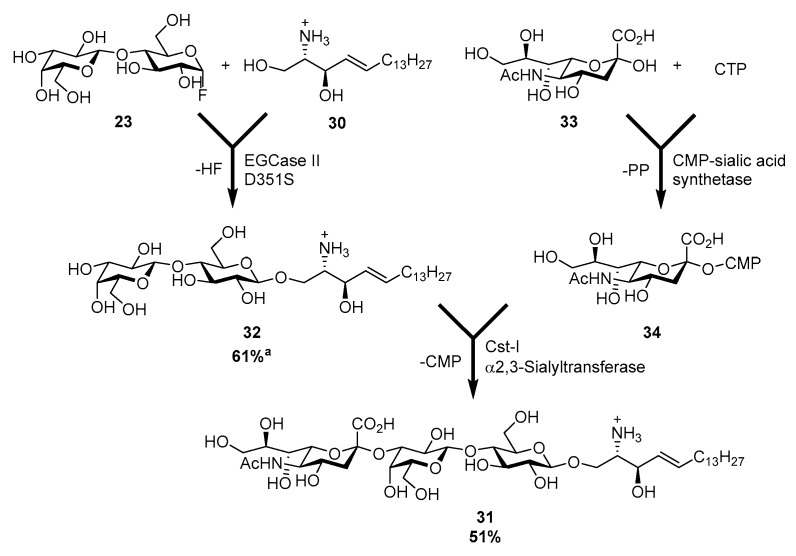
Synthesis of the glycolipid *lyso*-G_M3_ (**31**) by Rich et al. [36]. The production of the lactosyl sphingosine acceptor **32** for the Cst-I α-2,3-sialyltransferase was catalyzed by the EGCase II glycosynthase in an overall yield of 61%. ^a^ Yield encompassing the chemical synthesis of LacF (**23**) and the glycosynthase reaction.

Goddard-Borger et al. demonstrated the use of a glycosynthase in the synthesis of psychosine, a β-d-galactopyranosyl sphingosine found in the central nervous system [35]. Accumulation of this compound can lead to neural signaling dysfunction often observed in individuals with Krabbe disease. The β-glycosynthase EGALC E341S (*endo*-galactosyl ceramidase of *R. equi*) was successfully employed in the one step synthesis of psychosine from αGalF (**2**) and sphingosine (**30**) in a yield of 21%, the yield being limited mainly by problems involving the low solubility of sphingosine (**30**) and precipitation of the enzyme. Further glycosphingolipids were synthesized by the effective combination of glycosyltransferases and *endo*-glycoceraminidase (EGCase) derived synthases. Rich et al. described the synthesis of the glycolipid *lyso*-G_M3_ (**31**) by combining lactosyl fluoride (**23**) with sphingosine (**30**) catalyzed by EGCase II D351S and subsequent sialylation with the Cst-I α-2,3-sialyltransferase reaching an overall yield of 51% (**31**, Scheme 5) [36].

Yang et al. broadened the same synthesis to a FRET-probe of *lyso*-G_M3_ in order to visualize enzymatic processing of the glycolipid [37]. Another combination of glycosyltransferases and a glycosynthase was performed in sequential and also one-pot reactions by Henze et al. [38]. The report demonstrates the synthesis of *N*-acetyllactosamine type 1 (-3-Gal-β1,3-GlcNAc-1-) and type 2 (-3-Gal-β1,4-GlcNAc-1-) oligomers by combining the glycosynthase His_6_BgaC D233G (*B. circulans*) either in a one-pot or sequentially with the glycosyltransferases β3GlcNAcT (*H. pylori*) and the βGalT-1 (human origin) [39]. By sequential use of these enzymes, it was even possible to create neo-*N*-acetyllactosylamine (LacNAc) oligomers with alternating type 1 and type 2 units.

**Scheme 6:**
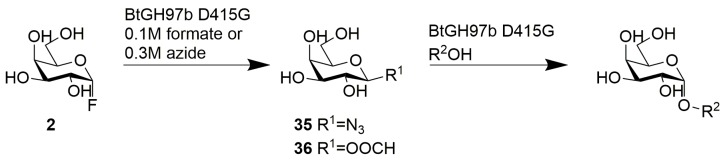
In situ formation of the glycosyl donors βGalN_3_ (**35**) or βGal formate (**36**) by incubation of αGalF (**2**) with glycosynthase BtGH97b D415G the additional external nucleophiles sodium azide of formate [41]. The in situ produced donor can then be subsequently transferred to a suitable acceptor (R^2^OH).

Even though much research and many advances have occurred in the synthesis of β-Gal linkages, the synthesis of α-Gal linkages utilizing α-galactosynthases is still limited. Cobucci-Ponzano et al. reported the effective conversion of β-d-galactopyranosyl azide (**35**, βGalN_3_) to α-galacto-oligosaccharides by the glycosynthase TmGalA D327G derived from *T. maritima* [40]. The enzyme produced galacto-oligosaccharides in good yields and the method was recently expanded by Okuyama et al. to the in situ formation of a β-d-galactosyl formate donor (**36**), which exhibited a higher transglycosylation rate compared to the azide donor (**35**, Scheme 6) [41]. The method allowed the galactosylation of carbohydrates such as glucose (α-1,1-β), xylose (α-1,4), maltose (α-1,1-β), cellobiose (α-1,1-β; α-1,6), lactose (α-1,1-β), and pNP derivatives of glucose (pNPGlc, **37**), and mannose (pNPMan, **6**; exclusively α-1,6) in yields ranging from 75 to 95%. Bayón et al. examined the addition of green co-solvents towards the synthesis of α-galactosyl residues with βGalN_3_ (**35**) and reported increased conversion and yield for the glycosynthase TmGalA D327G producing α-1,6 bonds with pNPGlc (**37**) and pNPMan (**6**) [42].

### 2.3. Xylosynthases

**Scheme 7:**
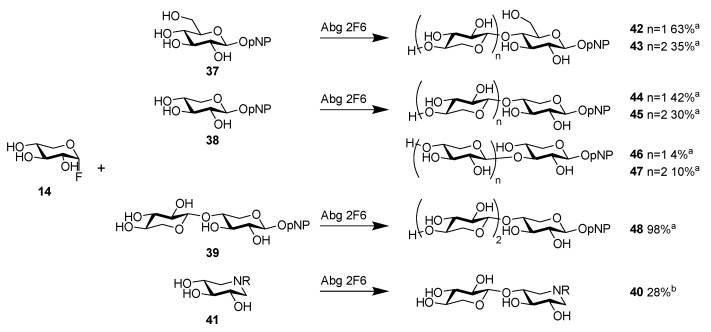
Synthesis of various xylosides **40**, **42**–**48** by the glycosynthase Abg 2F6 derived from the β-glucosidase Abg of *A. tumefaciens* [44]. The enzyme exhibited variable selectivity depending on the acceptor substrate, producing predominantly β-1,4 linkages. ^a^ Yields determined by HPLC analysis of the reaction mixture; ^b^ Yield of isolated, acetylated product.

Xylooligosaccharides have gained much interest in the pharmaceutical industry, due to their anti-freezing activity, non-digestibility, non-cariogenic and beneficial properties for the intestinal flora [43]. One of the first approaches to the synthesis of xylosides was carried out by Kim et al. using the β-glycosynthase Abg 2F6 (A19T, E358G, Q248R, M407V) derived from a glucosidase from *A. tumefaciens* [21]. The glycosynthase variant exhibited a broad donor substrate range and showed an increased specificity to α-d-xylosyl fluoride (αXylF, **14**; 34× higher) compared to the original mutant Abg E358G. This was then utilized in 2005 by the same group in the synthesis of xylosaccharides with pNPGlc (**37**, β-1,4), pNPXyl (**38**, β-1,3/1,4) and pNPXylobioside (**39**, Xyl_2_pNP; β-1,4) as acceptors in yields of 35–98%. Xylosylated 1-deoxyxylanojirimycin (**40**), a xylose derived nitrogen-containing inhibitor, was also synthesized by this mutant in a yield of 28% (acetylated product; Scheme 7) [44].

**Scheme 8:**
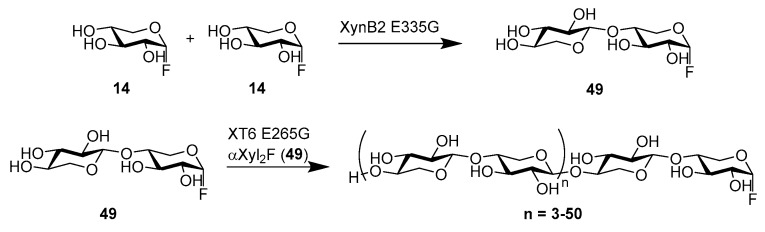
Production of xylooligomers comprising 6–100 monomers by exploitation of the self-condensation of αXylF (**14**) and Xyl_2_F (**49**) catalyzed by the enzyme combination of XynB2 E335G and XT6 E265G [48,49].

After these first syntheses, various groups accomplished the synthesis of xylooligomers with glycosynthases derived from actual xylanases. The reducing-end xylose-releasing *exo*-xylanase (REX) mutated in the acid/base catalytic residue (E236C) was utilized by Honda et al. in the synthesis of xylotrimers from xylobiosyl fluoride (X_2_F, **49**) and xylose [45]. This mutant enzyme was also the first report of a glycosynthase derived from an inverting glycosidase. Kim et al. also synthesized xylooligosaccharides with the CFXcd-E235G mutant of the retaining xylanase of *C. fimi* [46]. The enzyme catalyzed the oligomerization of X_2_F (**49**) and Xyl_2_pNP (**39**) or benzylthio-β-xylobioside (Xyl2BT) resulting in a mixture of oligomers ranging from xylotetrasaccharides to -dodecasaccharides with a total yield of transfer products of 61% and 66%, respectively. The Sugimura et al. group expanded the repertoire of xylanase originating glycosynthses by four examples of the GH10 family (XylB *T maritima*; XynA *B. halodurans*; XynB *C. stercorarium*; Cex *C. fimi*) enabling the synthesis of xylooligomers from X_2_F (**50**) in yields from 29–69% (7.8–18.4 mg) [47]. The products precipitated enabling easy isolation and were of high interest as unsubstituted xylan, a homopolymer of β-1,4-xylopyranose units, does not exist in nature. The production of larger xylooligomers was achieved by the group of Ben-David et al. [48]. The XynB2 E335G mutant of the retaining *exo*-glycosidase (GH52) of *G. stearothermophilus* catalyzed the synthesis of xylosides with αXylF (**14**) and pNPGlc (**37**), pNPXyl (**38**), and pNPMan (**6**) with a β-1,4 linkage in yields of 49%, 42% and 10% (disaccharide yields), respectively. By exploitation of the self-condensation of αXylF (**14**) by the enzyme to Xyl_2_F (**49**), a combinatorial synthesis with a glycosynthase of the xylanase XT6 (E265G; also of *G. stearothermophilus*) resulted in xylooligomer comprising of 6–100 monomers (Scheme 8) [48,49].

**Scheme 9:**
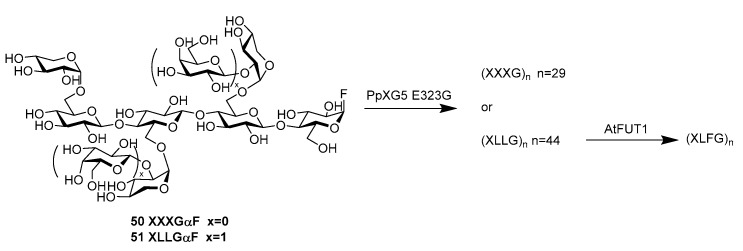
Combinatorial glycosynthase/glycosyltransferase approach for the production of defined, homogenous xyloglucans [53]. Polysaccharide synthesis using compound **50** or **51** was catalyzed by PpXG5 E323G resulting in polymers with a maximal molecular weight of 30,000 (*n* = 29) and 60,000 (*n* = 44), respectively. Subsequent fucosylation by AtFUT1 reached a fucosylation of 75% of the oligosaccharide repeats. Nomenclature: X = Xyl-α1,6-Glc; L = Gal-β1,2-Xyl-α1,6-Glc; F = Fuc-α1,2- Gal-β1,2-Xyl-α1,6-Glc.

The activity of the XynB2 E335G mutant in production of Xyl_2_F (**49**) and Xyl_3_F was improved by the development of a general screening assay utilizing the pH indicator Methyl Red, resulting in mutants with much higher k_cat_ values than the original synthase [50]. However, the yields of the reactions catalyzed by the improved mutants were not reported. More recently Goddard-Borger et al. described the effective synthesis of various xylanase inhibitors by the *exo*-β-xylosidase mutant Bhx E334G (*B. halodurans*) [51]. The enzyme accepted different sugar derivatives such as thioglycosides, iminosugar derived carbamates, and a 2-deoxy-2-fluoroxyloside giving yields of 67–86% of glycosylated products (acetylated products). A further important type of xyloside are xyloglucans (an α-1,4 glucan backbone containing α-1,6-d-xylose branches) which are the principal hemicellulose component of the primary cell wall of plant cells and are also an abundant type of storage polysaccharide in seeds [52]. They often find use in the food, paper, textile, and pharmaceutical industry as for example cellulose crosslinkers or rheology modifiers (in fluid mechanics). The synthesis of xyloglucans, as custom polysaccharides, was recently carried out by Spaduit et al. with the broad-specificity xyloglucan glycosynthase PpXG5 E323G (*P. pabuli*) [53]. The group synthesized XXXG- (**50**) and XLLG-structure (**51**) type xyloglucans of molecular masses up to 30,000 (*n* = 29) and 60,000 (*n* = 44) by self-condensation of XXXGαF (**50**) and XLLGαF (**51**) donor blocks respectively (Scheme 9). These were subsequently modified to fucosylated XLFG type glucans with a ratio of 3:1 XLFG:XLLG catalyzed by the fucosyltransferase AtFUT1(originating from *A. thaliana*).

### 2.4. Mannosynthases

**Scheme 10:**
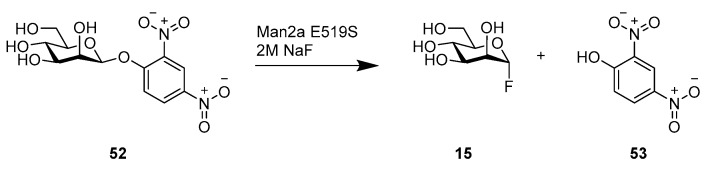
In situ production of the glycosylation donor αManF (**15**) by exploiting the chemical rescue of hydrolytic activity of Man2a E519S in the presence of sodium fluoride as an external nucleophile [54].

Next to the xyloglucans, mannans are also highly abundant polysaccharides in plant cell walls and mannosyl moieties are common components of many biological structures such as *N*-linked glycans, microbial and viral antigens [54,55]. The enzymatic synthesis of β-mannosyl linkages has gained much interest as the chemical synthesis is complex and demanding as there are no beneficial effects promoting the synthesis of this anomeric configuration. The use of β-mannosynthases is therefore a simple and practical solution for the synthesis of this type of bond. Nevertheless, only little research has been carried out toward the creation of mannosynthases. Nashiru et al. created a β-mannosynthase by the glycosynthase method with a retaining β-mannosidase mutant of Man2a (E519S, GH2) of *C. fimi* [54]. The enzyme catalyzed the formation of β-1,4 (predominant linkage) and β-1,3 mannosides with α-mannosyl fluoride (**15**, αManF) and various acceptors (pNPMan (**6**), -Xyl (**38**), -Glc (**37**), -cellobioside (**8**), -gentiobioside, and 2,4-dinitrophenyl-2-deoxy-2-fluoro-β-mannoside) in yields from 70–99% comprising di-, tri-, and tetrasaccharides. The yield was mostly hampered due to the instability of αManF (**15**), which can easily undergo hydrolysis by epoxide-formation. The enzyme also exhibited high activity in chemical rescue experiments using 2,4-dinitrophenyl-β-d-mannosylpyranoside (**52**) and external nucleophiles such as azide, formate or acetate, but also with fluoride ions allowing an in situ production of the αManF donor (**15**, Scheme 10).

Larger mannooligosaccharides were produced by Jahn et al. by biocatalysis using the glycosynthase derived from the β-mannosidase of *C. japonicus* Man26A [56]. The glycosynthase produced mannooligosaccharides using αMan_2_F as a donor and pNPGlc (**37**) as the acceptor in a total yield of 59%. The tri- (36%), penta- (18%), and heptasaccharides (5%) contained exclusively β-1,4 linkages. The acceptor pNPMan (**6**) on the other hand resulted in a trisaccharide in a 35% yield. In contrast to these syntheses in which oligomerization of the product is observed, Yamamoto et al. described the possibility of mono-glycosylating acceptors with a glycosynthase derived from a broad glycosidase scaffold [55]. The α-mannosynthase was created by mutation of an α-glucosidase of *S. solfataricus* with a preferred hydrolysis of glucosides over mannosides. The synthase variant MalA D320G catalyzed the formation of oligosaccharides if βGlcF was used, but only mono-glycosylation in the case of the βManF donor. The enzyme was tested on a variety of pNP-glycosides resulting in yields up to 77% (α-1,4 being the predominant type of linkage, α-1,3 and 1,2 were also observed) and the preparation of naturally occurring α-mannosyl motifs, such as Man-α(1,4)-Glc, Man-α(1,3)-l-Rha (**54**), and Man-α(1,2)-Man (**55**, Table 2).

The method also allowed the production of mannosylated *myo*-inositol (**56**/**57**) which is a component of the cell wall glycolipids of *Mycobacterium tuberculosis*. The mono-mannosylated inositols were isolated in a yield of 41% (mixture of Man-α(1,5)- and Man-α(1,1)-*myo*-inositol **56** and **57** in a 3:1 ratio).

### 2.5. Fucosynthases

A further important group of compounds relevant in many biological processes are fucosyl residues. This deoxy-aldohexose exists in nature, in comparison to most natural carbohydrates, as a l-configured glycoside and linked predominantly in α-configuration at the anomeric center. Many biological processes involve fucosylated compounds such as, cell-to-cell communication, cell development, recognition structures for pathogens, and antigenic structures.

**Scheme 11:**
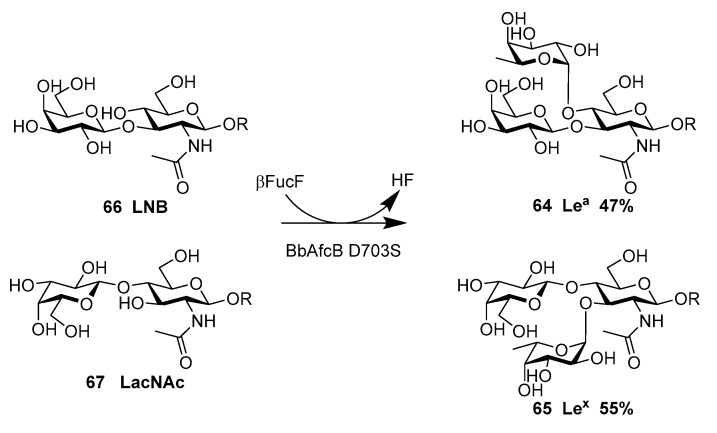
α-Fucosylation of LNB **66** or LacNAc **67** for the production of the Lewis antigens Le^a^ (**64**) and Le^x^ (**65**), respectively, catalyzed by the α-1,3-1,4-l-fucosynthase BbAfcB D703S [59].

The first synthesis of fucosylated structures using the glycosynthase method was reported by Wada et al. with a mutated α-fucosidase of *B. bifidum* BbAfcA D766G [57]. The enzyme was successfully utilized in the synthesis of a Fuc-α(1,2)-Gal linkage in 2′-fucosyllactose by combining β-fucosyl fluoride (βFucF) with lactose. However, the low stability of βFucF led to yields of 6%. The yields caused by the instability of the β-fluoride donor were overcome by Cobucci-Ponzano et al. by the implementation of more stable β-azide fucosylpyranoside (βFucN_3_) as donors for two new α-fucosynthases [58]. The α-fucosynthase SsD242S exhibited a wide acceptor range (shown for various aryl-glycosides) though also catalyzing the self-condensation of βFucN_3_ in all cases. In comparison, the second fucosynthase TmD224G demonstrated a more restricted acceptor range, but the self-condensation reaction was not observed. The reactions were also carried out with SsD242S in a preparative scale with total transfucosylation efficiencies ranging from 29–86% (for the acceptors pNPXyl (**38**), pNPGal, and pNPGlcNAc (**7**)) providing mostly α-1,4 and 1,3 linkages and additionally α-1,6 linkages in the case of the galactoside acceptor. The mutant TmD224G could even reach a transfucosylation efficiency of 91% producing a mixture of Fuc-α(1,3)- and Fuc-α(1,4)-β-XylpNP in a near 1:1 ratio. The application of α-l-fucosynthases was more recently transferred to relevant biological structures such as epitopes of the antigens Lewis and ABO. Sakurama et al. reported the synthesis of the lewis antigens Le^a^ and Le^x^ (Gal-β1,3/4-(Fuc-α1,4/3)-GlcNAc: Le^a/x^ **64**/**65**) catalyzed by the 1,3-1,4-α-l-fucosynthase BbAfcB D703S [59]. The antigen structures were created by the reaction of βFucF and lacto-*N*-biose I (**66**, LNB, Gal-β(1,3)-GlcNAc) and *N*-acetyllactosamine (**67**, LacNAc, Gal-β(1,4)-GlcNAc) resulting in yields of 47% and 55% for Le^a^ (**64**) and Le^x^ (**65**), respectively (Scheme 11).

An increase in yield using the more stable donor βFucN_3_ as demonstrated by Cobucci-Ponzano et al. could not be achieved as no transfucosylation products could be observed with this donor. The enzyme also interestingly only acted on di- or trisaccharide acceptors unable to fucosylate monosaccharides such as, Glc, Ga**l**, GlcNAc, and GalNAc. The specific production of lacto-*N*-fucopentaose II (LNFP II: Gal-β1,3-(Fuc-α1,4)-GlcNAc-β1,3-Gal-β1,4-Glc) was also achieved in a yield of 41% with lacto-*N*-tetraose (LNT: Gal-β1,3-GlcNAc-β1,3-Gal-β1,4-Glc) as the acceptor.

**Scheme 12:**
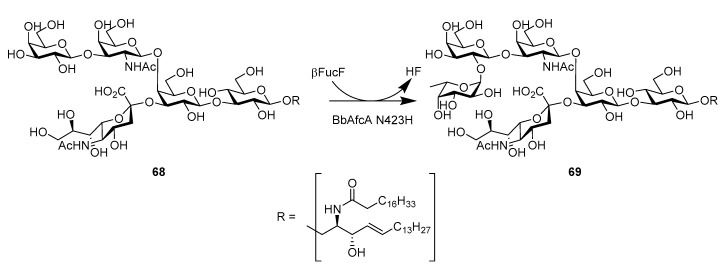
Selective introduction of an α-fucosyl residue to the glycoside of the GM1 ganglioside (**68**) demonstrated by Sugiyama et al. [61]. The enzyme BbAfcA N423H showed a broad acceptor range exhibiting fucosylation activity also towards *N*- and *O*-glycans and a nonasaccharide xyloglucan.

The repertoire of synthesized antigen epitope structures was expanded by the work of Sugiyama et al. who introduced the 1,2-α-l-fucosynthase BbAfcA N423H [60]. In addition to fucosylating many types of mono- and disaccharides, producing for example H type-1 or H type-2 chains from LNB (**66**) or LacNAc (**67**) respectively, the antigens Le^b^ and Le^y^ (Fuc-α1,2-Gal-β1,3/4-(Fuc-α1,4/3)-GlcNAc: Le^b/y^) were produced in yields of 43% and 62% from the Le^a^ and Le^x^ trisaccharides (**64** and **65**). In comparison to the BbAfcB D703S fucosynthase, BbAfcA N423H produced LNFP I (Fuc-α1,2-Gal-β1,3-GlcNAc-β1,3-Gal-β1,4-Glc) rather than LFNP II with an efficiency of 75%. Subsequently, the transfer of fucosyl residues to the non-reducing end of *O*-linked glycans was demonstrated by the successful reintroduction of H-antigen structures to pretreated glycopeptide porcine gastric mucin. The application was even further extended by the group introducing H-antigens to the non-reducing ends of *N*- and *O*-glycans in fetuin glycoproteins, GM1 ganglioside (**68**), and a xyloglucan nonasaccharide [61]. It was demonstrated that BbAfcA N423H could fucosylate asialo-bi-, asialo-tri-, and monosialo-tri-antennary *N*-glycan of the asialo fetuin glycopeptide with transfer efficiencies of 9%, 26%, and 20% respectively. It was also possible to fucosylate the sialo form of the fetuin peptide containing di-sialo-bi-, di-sialo-tri, tri-sialo-tri, and tetra-sialo-tri-antennary glycans, yet with lower transfer efficiencies. The fucosylation of the xyloglucan nonasaccharide XLLG (**51**, Structure shown in Scheme 9) occurred with an efficiency of 57% resulting in a mixture of mono- (α-1,2-Gal), di- (α-1,2-Gal), and trifucosylated (α-1,3-Glc) XLLG glycosides. The enzyme also fucosylated glycolipids, transferring α-1,2-fucosyl residue to the glycoside of the GM1 ganglioside (**68**, Scheme 12).

### 2.6. Glycosynthase Variants of endo-β-N-Acetylglucosaminidases

**Scheme 13:**
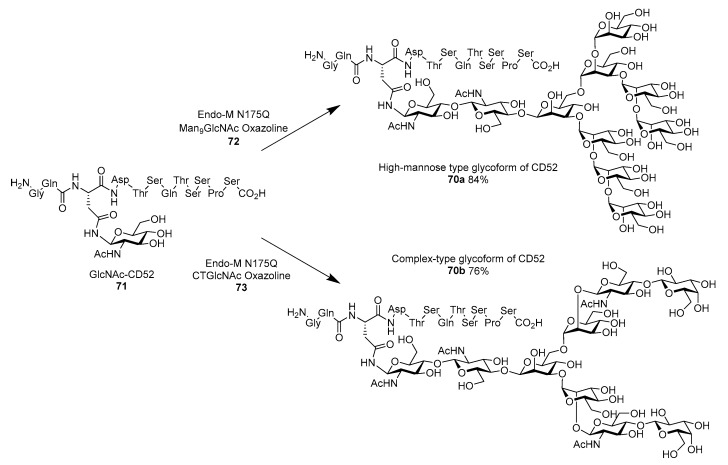
Synthesis of two glycoforms of the sperm antigen CD52 (**70a**/**b**) by the *endo*-β-*N*-acetylglucosaminidase derived glycosynthase Endo-M N175Q [65]. The high-mannose and complex type glycoforms were produced under utilization of oxazoline donors **72**, **73** and the GlcNAc-CD52 peptide (**71**).

Different from the *exo*- and *endo*-glycosidases described above, the hydrolytic mechanism of *endo*-β-*N*-acetylglucosaminidases follows a substrate-assisted pathway in which the anomeric center of the *N*-acetylglucosamine located in the chitobiose moiety undergoes a nucleophilic attack by the *N*-linked acetyl group creating a oxazoline structure. This kind of activated structure can be employed by mutant forms of the *endo*-β-*N*-acetylglucosaminidases, which are deficient in the promotion of the oxazoline formation, as glycan donors in glycosynthetic reactions. The first glycosynthase like form of an *endo*-β-*N*-acetylglucosaminidase was reported by Umekawa et al. as the N175A variant of Endo-M (originating from *M. hiemalis*) [62]. The enzyme could utilize the oxazoline donor Man_9_GlcNAc-oxazoline in the synthesis of the HIV-1 gp41 glycopeptide Man_9_GlcNAc_2_C34, producing the glycopeptide in a yield of 72%. A large improvement for the glycosynthase method using *endo*-β-*N*-acetylglucosaminidases was the simplified donor synthesis by Noguchi et al. [63,64]. The synthesis of the oxazoline structure catalyzed by 2-chloro-1,3-dimethyl-imidazolium chloride (DMC) and later 2-chloro-1,3-dimethyl-1*H*-benzimidazol-3-ium chloride (CDMBI) allowed the production in aqueous solution in unprotected form, therefore ideal for subsequent conversion by enzymatic glycosylation. In 2010, Umekawa et al. described a new variant Endo-M N175Q, which exhibited a much higher synthase activity and transglycosidase activity [65]. This enabled the use of natural glycan donors such as the sialoglycopeptide (SGP), though leading to a lower yield compared to the use of oxazoline donors. The variant enabled the synthesis of a high-mannose (84%) and complex type glycoform (76%) of the sperm antigen CD52 (**70a**,**b**) using the GlcNAc-CD52 peptide (**71**) as the acceptor (Scheme 13). The group also described the successful synthesis of sialo-complex-type glycoforms of the bioactive peptides PAMP12 and Substance P in yields of 95 and 98%, respectively. The glycosylation reaction was also shown for the protein RNaseB bearing a single GlcNAc moiety therefore allowing glycan modification of proteins/enzymes by this method [66]. Amin et al. also described the production of monoglycoforms of RNaseB by the glycosynthase Endo-A N171A (*A. protophormiae*) in yields up to 80% with chemically synthesized oxazoline glycans [67].

The Endo-M and Endo-A glycosynthase variants were additionally employed in the synthesis of four glycoforms (yields varying from 59–96%) of the glycopeptide pramlintide by Tomabechi et al. in an attempt to improve the low circulatory half-life and poor solubility of the pharmaceutical compound [68]. The produced glycoforms were tested in vitro and in vivo and exhibited varying properties in dependency of the type of transferred glycan and its position in the glycopeptide. The glycoform library was subsequently expanded with the same enzymes by Kowalczyk et al. to 18 *N*-glycosylated pramlintide analogues bearing either a GlcNAc-, pentasaccharide-, or undecasaccharide residue at different positions of the peptide [69]. In comparison to the Endo-A and Endo-M glycosynthases, Fan et al. developed a glycosynthase variant of Endo-D originating from *S. pneumoniae* which could glycosylate fucosylated GlcNAc residues [70]. This unique property allowed the remodeling of the glycans of the IgG Fc-domain. However, the strict substrate specificity for Man_3_GlcNAc oxazoline and not complex type *N*-glycan oxazolines limits the use of the enzyme greatly. A more variable glycosylation of α-1,6-fucosylated GlcNAc-polypeptides was demonstrated by Giddens et al. with the Endo-F3 mutants D165A/Q [71]. The mutant was capable of synthesizing asialo biantennary and complex triantennary core-fucosylated glycoforms of rituximab (intact antibody) in yields over 95%. Further applications, indicating the high potential of the method for the synthesis of pharmaceutical relevant compounds were the chemoenzymatic production of vaccine candidates [72]; the site-selective glycosylation of HIV-1 polypeptide antigen bearing two different glycans (yields up to 95%) [73]; the glycan remodeling of human erythropoietin (EPO) [74]; and the synthesis of mannose-6-phosphate-containing glycoproteins [75]. Tang et al. impressively demonstrated a one-pot *N*-glycan remodeling of IgG proteins by combining the wild type (*wt*) Endo-M glycosidase with the synthase variant Endo-S D322S [76]. This synthesis comprised the donor production by Endo-M catalyzed SGP hydrolysis, subsequent conversion to the oxazoline with DMC, and donor transfer to the protein by Endo-S D322S with near complete conversion within 30 min. Further yield improvement might be achieved by the new glycosynthase of Endo-CC (*C. cinereal*) recently introduced by Eshima et al. [77]. The enzyme exhibited high activity at a neutral pH of 7.5 in comparison to the acidic pH between 4–6, which most other *endo*-β-*N*-acetylglucosaminidases require for activity. This could be a great advantage for yield improvement due to the increased stability of the oxazoline donors at this pH [78]. A further use of the oxazoline type donor for glycoside synthesis has been demonstrated by the production of glycosaminoglycans such as derivatives of chondroitin sulfate and hyaluronan [79,80,81,82]. However, the polymerization of the oxazolines derived from, for example *N*-acetylchondrosine, was carried out with the natural forms of ovine or bovine testes hyaluronidase, therefore not utilizing the glycosynthase method and not further discussed in this review. Nevertheless, the first step to glycosynthase mediated synthesis of glycosaminoglycans such as heparan, chondroitin sulfate, and hyaluronan was demonstrated by Müllegger et al. by the synthesis of uronic acid-containing glycoconjugates catalyzed by the thermostable glycosynthase derived from *T. maritima* β-glucuronidase [83].

## 3. Conclusions

The enzymatic synthesis of glycosidic structures utilizing the glycosynthase approach has made many advances and is nowadays a promising alternative to classical chemical synthesis. The direct comparison of enzymatic glycosylation reactions compared to the chemical paths is difficult due to the additional protection and deprotection as well as possible modification steps needed after chemical glycosylation. When solely comparing the glycosylation reaction, the chemical pathway also in many cases produces glycosides in similar high yields (e.g., the synthesis of disaccharide **61** (Section 2.4, Table 2), enzymatic glycosylation 55% yield; chemical glycosylation 61%). However, when considering the steps required for synthesis of the acceptor and donor molecule and also the subsequent deprotection steps for the chemical pathway, the advantage of the glycosynthases becomes obvious, especially when thinking of large oligosaccharides (Table 3).

The repertoire of glycosynthase variants is successfully increasing though it is still insufficient for creating the high diversity of glycosides present throughout Nature. Great advances were acheived with the introduction of synthases derived from inverting enzymes and the recent glycosynthases derived from *endo*-β-*N*-acetylglucosaminidases that allow the production of peptides with defined glycosylation. A larger drawback, however, is still the lack of glycosynthase variants capable of glycosylating non-glycosidic structures and the formation of oligomerized side products.

Further production of glycosynthase variants of glycosidases with known substrates will be vital for the progress of this method. Also progress in methods for identification and characterization of glycosynthases from mutant libraries will be critical to produce enzymes with new acceptor or donor scopes. Many advances have already been made such as biological and biochemical assays utilizing chemical complementation, fluorescence and photometric methods [50,88,89,90]. Subsequent analysis of structural and mechanistic details will help to identify structural requirements, which can be transferred to new glycosynthases by genetic engineering and rational design [28,91].

## Figures and Tables

**Table 1:** Examples of synthetic glycosylation products catalyzed by the TnG E338A [22]. Reactions were carried out with α-d-GlcF (**1**), α-d-GalF (**2**), or α-d-FucF (**3**) as the donor. Donor and acceptor molecules were employed in equal amounts unless otherwise noted. Yields were determined after purification or isolation.

Donor	Acceptor	Product Yield (%)
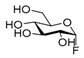 **1**	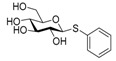 **4**	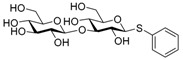 **9** 80%
	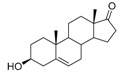 **5**	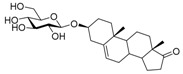 **10** 49%
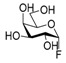 **2**	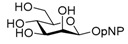 **6**	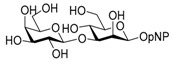 **11** ^a^ 14%
	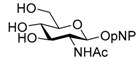 **7**	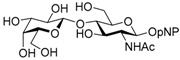 **12** 32%
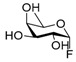 **3**	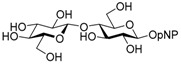 **8**	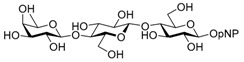 **13** 91%

^a^ Donor was used in 2 equivalents.

**Table 2:** Synthetic products of α-mannosylation catalyzed by the MalA D320G [55]. All reactions were carried out with βManF as the donor. Different regioselectivities were observed depending on the acceptor structure and anomeric configuration. Yields were determined after purification or isolation.

Acceptor	Product		Yield (Selectivity)
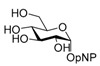 **37**	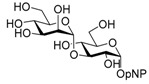 **60**		26% ^a^ (>95%, α-1,3)
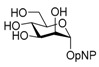 **6**	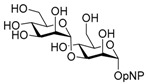 **61**	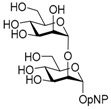 **62**	55% (60:40 α-1,3/α-1,2)
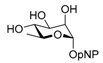 **58**	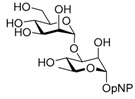 **63**		44% ^a^ (92%, α-1,3)
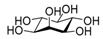 **59**	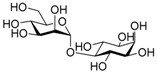 **56**	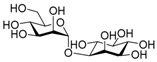 **57**	41% ^a^ (3:2 α-1,5/α-1,1)

^a^ Isolated as peracetate.

**Table 3:** Comparison of the required steps and yields of the chemical and enzymatic synthesis of the disaccharide Man-α(1,3)-d-α-pNPMan (**61**) [55,84,85,86,87].

Type	Donor	Acceptor	Product (Yield, Selectivity)	References
Chemical	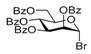 **74** 2 Steps 83%	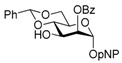 **75** 2 Steps 50% ^a^	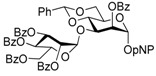 **76** Glycosylation 61% Deprotection of **76** 81% (2 Steps 49%)	[84,85,86]
Enzymatic	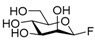 **15** 2 Steps 21% ^b^	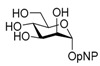 **6** commercially available	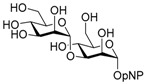 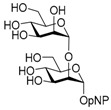 **61 62** 55% (60:40 α-1,3/α-1,2)	[55,87]

^a^ Starting from commercially available α-pNPMan (**6**); ^b^ Starting from 2,3,4,6-tetra-*O*-acetyl-β-d-mannopyranose.
